# Versatile small molecule kinase assay through real-time, ratiometric fluorescence changes based on a pyrene-DPA-Zn^2+^ complex†

**DOI:** 10.1039/d1ra01547h

**Published:** 2021-03-10

**Authors:** Jihoon Kim, Jinyoung Oh, Min Su Han

**Affiliations:** Department of Chemistry, Gwangju Institute of Science and Technology (GIST) 123 Cheomdangwagi-ro, Buk-gu Gwangju 61005 Republic of Korea happyhan@gist.ac.kr

## Abstract

A real-time kinase assay method based on a ratiometric fluorescence probe that can be applied to various small-molecule kinases is described herein. The probe can trace the reversible interchange of ATP and ADP, which is a common phenomenon in most small-molecule kinase reactions, by a ratiometric fluorescence change. This property facilitates the monitoring of phosphorylation and dephosphorylation in small-molecule kinases, whereas most of the existing methods focus on one of these reactions. To prove the applicability of this method for small-molecule kinase assays, hexokinase and creatine kinase, which phosphorylate and dephosphorylate substrates, respectively, were analyzed. The ratiometric fluorescence change was correlated with the enzyme activity, and the inhibition efficiencies of the well-known inhibitors, *N*-benzoyl-d-glucosamine and iodoacetamide, were also monitored. Notably, the change in fluorescence can be observed with a simple light source by the naked eye.

## Abstract 2

A versatile assay system that can be trace both phosphorylation and dephosphorylation by small molecule kinase is demonstrated, and can be applied regardless of substrate diversity.

## Graphical abstract

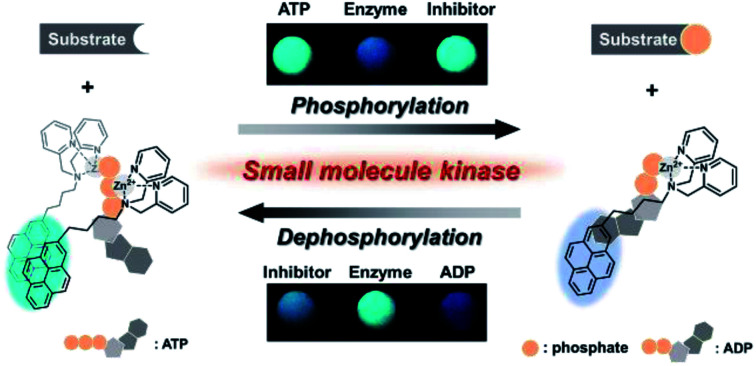

## Introduction

Kinases mediate the transfer of a phosphate group to an acceptor for phosphorylation or dephosphorylation of various substrates. This enzymatic reaction is related to a number of biological processes, including metabolism, transcription, cell cycle regulation, and apoptosis.^1–4^ Because the dysregulation of kinase enzymes can cause various human diseases, the development of kinase assay methods and inhibitors for each kinase plays a critical role in the diagnosis and therapy of these diseases.^5–10^ Although many kinase assay methods have been reported for the rapid detection of enzyme malfunctions and inhibitor screening, most of them have focused on protein kinases, despite the importance of small molecule kinases, which utilize small molecules as their substrates. Many protein kinase assay methods have been established by using the characteristic properties of the protein substrates, and the dynamic range of the substrates was also suitable for proteins.^11–14^ For specific small molecule kinase assays, examples have been developed by using nanoparticles, and coupled enzymes. Unfortunately, the enzyme assays showed obvious drawbacks in their end-point measurement and complex cascade enzyme reaction. Most of them also attempted to target unique structures of substrates that were unsuited for other crucial kinases.^15–19^ Thus, it remains necessary to develop a small molecule kinase assay method that can be applied in real time and is based on a property shared by most small molecule kinases, regardless of their substrate diversity.

In many small molecule kinases, a decrease in ATP, accompanied by an increase in ADP, commonly occurs, and this aspect has received attention as a target for monitoring kinase activity.^20–25^ However, some small molecule kinases have displayed an increase in ATP and a decrease in ADP for dephosphorylation of the substrate, unlike those kinases using ATP as a phosphate group donor. For example, creatine kinase (CK), which plays an important role in cellular energy transformation, is an example of a kinase that can perform reversible reactions. Mitochondrial CK catalyzes the transformation of creatine and ATP to phosphocreatine and ADP, whereas the production of creatine and ATP in the cytosol is caused by cytosolic CK to maintain energy homeostasis.^26,27^ Therefore, to understand small molecule kinases more precisely, it is necessary to monitor the reversible changes, rather than the mono-directional transformation. For this reason, it is proposed that real-time assay to trace the reversibly changed ATP and ADP ratio, which is a common feature of small molecule kinases, can be adopted as a general assay method that will be applicable to various small molecule kinases.

Ratiometric fluorescence probes have received attention because they allow for ratio change between emission bands at distinct wavelength and built-in correction to reduce environmental interference and improve sensitivity and accuracy, permitting the development of detection methods for various targets.^28–30^ In particular, certain ratiometric fluorescence probes have been introduced as a method for tracing reversibly changed analytes based on the significant property that the change of analytes can be monitored directly by the change of fluorescence emissions.^31,32^ For instance, the real-time interchange of bisulfate and hydrogen peroxide in a biological redox cycle was monitored by a ratiometric change of fluorescence emission.^32^ Considering the properties of ratiometric fluorescence probes, they could be effective for detecting bidirectional changes between ATP and ADP in a kinase reaction, and thus, they could become an appropriate real-time assay platform for various small molecule kinases associated with phosphorylation or dephosphorylation.

**Scheme 1:**
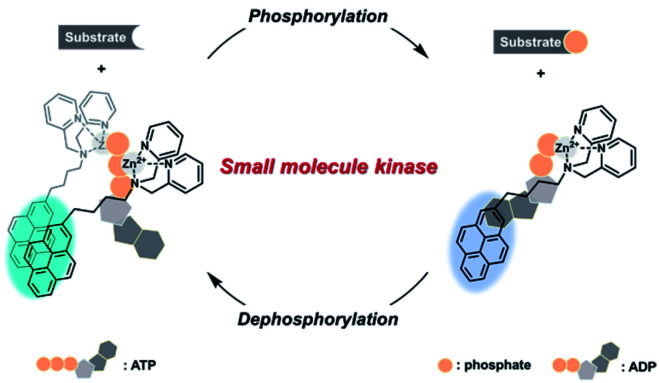
Schematic illustration of the real-time assay method for various small molecule kinases based on a ratiometric fluorescence probe.

In this study, a general method for a real-time small molecule kinase fluorescence assay was proposed. To develop the assay, a pyrene-dipicolylamine (DPA)-Zn^2+^ complex was prepared because this probe allowed discrimination between ATP and ADP with distinct ratiometric fluorescence emissions.^33,34^ It was speculated that a ratiometric fluorescence change in the pyrene-DPA-Zn^2+^ complex would occur in conjunction with the interchange of ATP and ADP, representing the real-time progression of the small molecule kinase reaction (Scheme 1). In addition, the reversible fluorescence change of the pyrene-DPA-Zn^2+^ complex would facilitate monitoring of the dephosphorylation as well as phosphorylation of the substrate by various small molecule kinases. As proof of this concept, the activity of two small molecule kinases, namely hexokinase (HK) to phosphorylate glucose and CK to dephosphorylate phosphocreatine, were analyzed, and inhibitor analyses for the enzymes were also demonstrated.

## Experimental

### Materials and instruments

Chemical reagents were purchased from commercial sources and, unless otherwise stated, were used without further purification. For enzyme assay, hexokinase (HK, EC 2.7.1.1) and creatine kinase (CK, EC 2.7.3.2) were purchased from Sigma Aldrich. Florescence spectra were recorded on an Agilent Cary Eclipse fluorescence spectrometer using a 1 cm path length quartz cell. ^1^H NMR spectrums were recorded on a JEOL 400 MHz NMR spectrometer.

### Synthesis

**Scheme 2:**
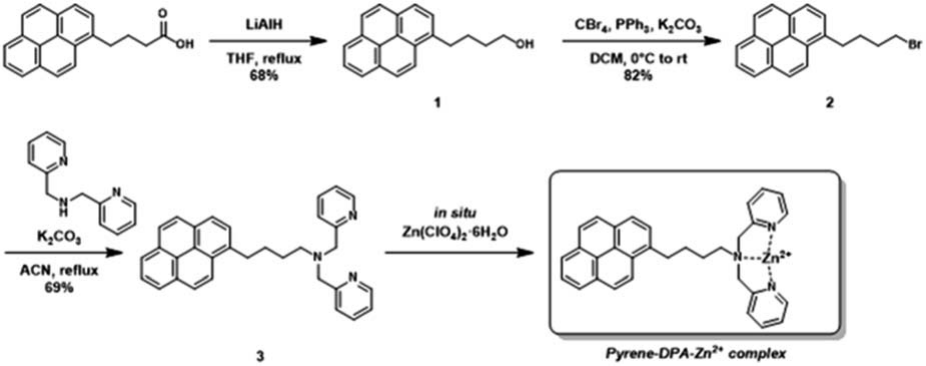
Synthetic route for preparation of pyrene-DPA-Zn^2+^ complex.

The pyrene-DPA-Zn^2+^ complex was prepared following as Scheme 2.

#### Synthesis of 1-pyrenebutanol (1)

1-Pyrenebutyric acid (1.00 g, 3.45 mmol) in anhydrous THF (22 mL) was stirred at 0 °C. To the solution lithium aluminum hydride (1.25 g, 32.5 mmol) dissolved in anhydrous THF (32.5 mL) was added dropwise slowly and stirred for 30 min. After then, the solution was refluxed for 23 h and quenched through Fieser method. The residue was evaporated and purified *via* silica gel column chromatography (MeOH : CHCl_3_ = 1 : 99) to give a pale yellow solid (0.64 g, 68%). ^1^H-NMR (400 MHz, CDCl_3_) *δ* 8.27 (d, *J* = 9.2 Hz, 1H), 8.16 (d, *J* = 2.1 Hz, 1H), 8.14 (d, *J* = 2.4 Hz, 1H), 8.11–8.08 (m, 2H), 8.02–7.96 (m, 3H), 7.86 (d, *J* = 7.9 Hz, 1H), 3.72–3.68 (m, 2H), 3.37 (t, *J* = 7.8 Hz, 2H), 1.97–1.90 (m, 2H), 1.77–1.70 (m, 2H) 1.25 (s, 1H).

#### Synthesis of 1-(4-bromobutyl)pyrene (2)

To 4-pyrenyl-1-butanol (0.500 mg, 1.82 mmol) solution in anhydrous DCM (30 mL) was added CBr_4_ (1.212 g, 3.64 mmol), and K_2_CO_3_ (0.504 g, 2.73 mmol) at 0 °C. PPh_3_ (0.716 mg, 2.73 mmol) in anhydrous DCM (20 mL) was added dropwise to the solution and stirred at room temperature for 4 h. After the reaction mixture was concentrated under reduced pressure, the residue was dissolved with EA and washed with water, brine, and dried on anhydrous Na_2_SO_4_, and concentrated under reduced pressure. The residue was purified by silica gel column chromatography (Hex : DCM = 7 : 1) to give a white solid (0.506 g, 82%). ^1^H-NMR (400 MHz, CDCl_3_) *δ* 8.27 (d, *J* = 9.2 Hz, 1H), 8.18–8.11 (m, 4H), 8.05–7.97 (m, 3H), 7.86 (d, *J* = 7.9 Hz, 1H), 3.49–3.46 (m, 2H), 3.40–3.36 (m, 2H), 2.05–2.02 (m, 4H).

#### Synthesis of [(2,2′-dipicolylamino)methyl]pyrene (3)

To 1-(4-bromobutyl)pyrene (0.100 g, 0.297 mmol) solution in ACN, 2,2′-dipicolylamine (0.071 g, 0.36 mmol), and K_2_CO_3_ (0.147 g, 1.07 mmol) was added and refluxed for 28 h. After evaporating solvent under reduced pressure, the residue was diluted with water and extracted with DCM. The collected organic layer was concentrated and purified by silica gel column chromatography (EA) to give a pale yellow oil (0.093 g, 69%). ^1^H-NMR (400 MHz, CDCl_3_) *δ* 8.49 (d, *J* = 4.6 Hz, 2H), 8.22–8.14 (m, 3H), 8.08–7.95 (m, 5H), 7.78 (d, *J* = 7.6 Hz, 1H), 7.55 (td, *J* = 7.6, 1.8 Hz, 2H), 7.46 (d, *J* = 7.6 Hz, 2H), 7.08 (ddd, *J* = 7.2, 5.0, 1.1 Hz, 2H), 3.79 (s, 4H), 3.26 (t, *J* = 7.6 Hz, 2H), 2.63 (t, *J* = 7.2 Hz, 2H), 1.88–1.80 (m, 2H), 1.74–1.67 (m, 2H).

#### *In situ* preparation of pyrene-DPA-Zn^2+^ complex

Pyrene-DPA-Zn^2+^ complex was generated *in situ* by addition of Zn(ClO_4_)_2_ to the buffer solution (HEPES, 20 mM, pH 7.4) containing compound 3. Without further incubation of the solution, other components were also added to the solution, and fluorescence spectra were recorded with excitation at 341 nm.

### Fluorescence measurement

#### Model study for real-time fluorescence kinase assay

To buffer solution (HEPES, 20 mM, pH 7.4) containing pyrene-DPA-Zn^2+^ complex (20 μM), different ratio of ATP and ADP (total 10 μM) were in 1 mL of volume. Emission spectra (*λ*_ex_ = 341 nm) were recorded using fluorescence spectrometer.

#### Monitoring phosphorylation in a kinase reaction

To buffer solution (HEPES, 20 mM, pH 7.4) containing pyrene-DPA-Zn^2+^ complex (20 μM), ATP (10 μM), MgCl_2_ (80 μM), and glucose (1 mM), various concentrations (0 to 1 unit) of HK were added. The fluorescence intensity was measured at 1 min interval for 30 min with excitation at 341 nm. The observed rate (*k*_obs_) for HK was estimated using initial change of fluorescence ratio of F_376_ and F_476_ from 0 to 5 min.

#### Kinetic study of hexokinase for glucose

Various concentrations of glucose (from 0 to 1.4 mM) was added to the buffer solution (HEPES, 20 mM, pH 7.4) containing pyrene-DPA-Zn^2+^ complex (20 μM), ATP (10 μM), MgCl_2_ (80 μM). After addition of HK (1 unit), the fluorescence intensity was measured at 1 min interval for 10 min with excitation at 341 nm. The Michaelis–Menten constant (*K*_m_), maximum initial velocity (*V*_max_) as the enzymatic parameters were estimated from Lineweaver–Burk plots and the values were compared with reported results.

#### Monitoring dephosphorylation in a kinase reaction

To buffer solution (HEPES, 20 mM, pH 7.4) containing pyrene-DPA-Zn^2+^ complex (20 μM), ADP (10 μM), MgCl_2_ (80 μM), and phosphocreatine (0.75 mM), various concentrations (0 to 1 unit) of CK were added. The fluorescence intensity was measured at 1 min interval for 15 min with excitation at 341 nm. The observed rate (*k*_obs_) for HK was estimated using initial change of fluorescence ratio of F_476_ and F_376_ from 0 to 4 min.

#### Inhibitor assay for the kinases

To buffer solution (HEPES, 20 mM, pH 7.4) containing pyrene-DPA-Zn^2+^ complex (20 μM), ATP (10 μM), MgCl_2_ (80 μM), and glucose (1 mM), a various concentration of *N*-benzoyl-d-glucosamine (0 to 4 mM), and without incubation HK (1 unit) was added. Inhibition efficiency was obtained by the change of excimer and monomer emission ratio at 20 min after the measurement.

To buffer solution (HEPES, 20 mM, pH 7.4) containing pyrene-DPA-Zn^2+^ complex (20 μM), ADP (10 μM), MgCl_2_ (80 μM), and phosphocreatine (0.75 mM), mixture with various concentrations of iodoacetamide (0 to 100 μM) and CK (1 unit per mL) incubated for 10 min was added. Inhibition efficiency was obtained by the change of excimer and monomer emission ratio at 15 min after the measurement.

## Results and discussion

### Model study for real-time fluorescence kinase assay

**Fig. 1:**
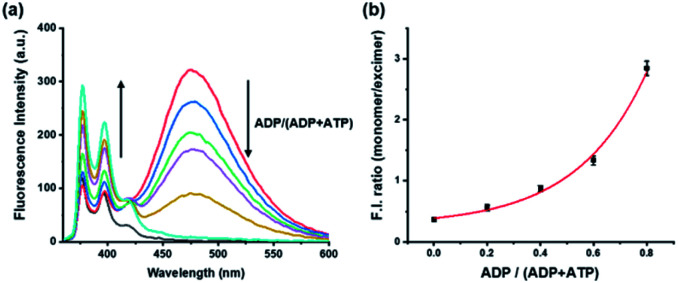
(a) Fluorescence spectra of pyrene-DPA-Zn^2+^ complex with various ratios of ADP and ATP in buffer solution (HEPES, 20 mM, pH 7.4); (b) fluorescence intensity ratio of monomer (F_376_) and excimer (F_476_) emission against the ratio of ADP and ATP. [pyrene-DPA-Zn^2+^ complex] = 20 μM, [ADP] + [ATP] = 10 μM, *λ*_ex_ = 341 nm.

To evaluate the influence on the pyrene-DPA-Zn^2+^ complex of ATP and ADP, which are common components for general kinase reactions, the fluorescence changes with ATP and ADP were measured. Addition of ADP to the pyrene-DPA-Zn^2+^ complex increased the monomer emission at 376 nm in proportion to the added concentration, whereas the excimer emission at 476 nm increased gradually as the concentration of ATP increased without a significant change in the monomer emission (Fig. S1†). As a result, ATP and ADP clearly had different effects on the pyrene-DPA-Zn^2+^ complex and induced two distinct pyrene complexes, as represented by the diverse emission spectra. In fact, it is postulated that the kinase reaction induces an interchange of ATP and ADP, not the fluctuation of each molecule, which may be the result of an increase in the ADP/ATP ratio for phosphorylation or decrease in the ratio for dephosphorylation. Therefore, the fluorescence change following a change in the ADP/ATP ratio was evaluated as a model study for monitoring kinase reactions. As shown in Fig. 1, a sequential change of the ADP/ATP ratio transformed the overall spectra, with an increase or decrease in the excimer emission accompanied by the opposite change in the monomer emission. This means that the interchange of ADP and ATP as a progression of the kinase reaction can be monitored in real time from the ratiometric fluorescence change.

### Monitoring phosphorylation in a kinase reaction and its kinetic study

**Fig. 2:**
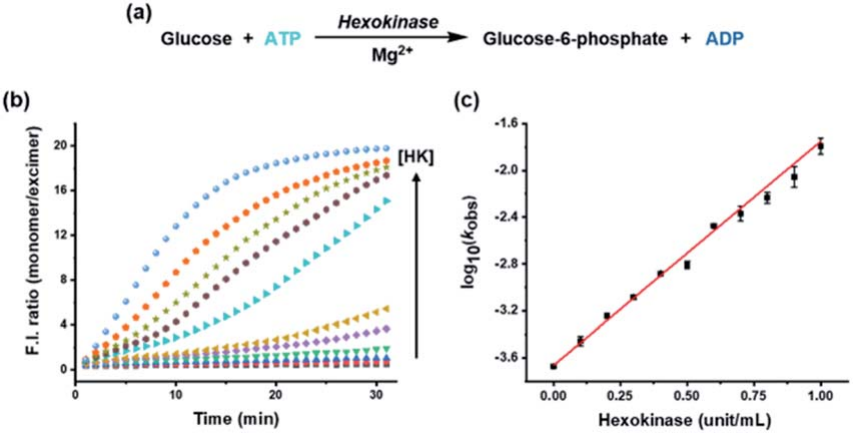
(a) Enzymatic reaction formula of HK associated with phosphorylation; (b) fluorescence intensity ratios of monomer (F_376_) and excimer (F_476_) emissions over time for the pyrene-DPA-Zn^2+^ complex with various concentrations of HK (0 to 1 unit) in buffer solution (HEPES, 20 mM, pH 7.4); (c) plot of the logarithm of *k*_obs_ against concentration of HK. [pyrene-DPA-Zn^2+^] = 20 μM, [ATP] = 10 μM, [Mg^2+^] = 80 μM, [glucose] = 1 mM, *λ*_ex_ = 341 nm.

To apply the system for assaying a biological kinase that phosphorylates its substrates, HK was analyzed as a model enzyme. HK, which plays an important role in the first step of glycolysis and has been well studied because of its relationship with many diseases, transfers a phosphate group from ATP to the substrate glucose, thereby increasing the ADP/ATP ratio (Fig. 2a).^35–37^ As the HK reaction proceeded and the ADP/ATP ratio increased gradually, the excimer emission at 476 nm gradually decreased with a concomitant increase in monomer emission at 376 nm, in conjunction with an increase in the concentration of HK from 0 to 1 unit (Fig. S2†); the monomer/excimer emission ratio changed in proportion to the concentration of HK during the reaction time (Fig. 2b). In addition, the logarithm of observed rate constant (*k*_obs_) for the enzyme reaction measured by using the ratiometric fluorescence change showed a linear correlation with the concentration of HK (Fig. 2c) and enzymatic parameters (*K*_m_, *V*_max_) obtained from the method showed little difference with reported value (Fig. S3, Table S1†), allowing quantification and kinetic study of the enzyme from the fluorescence change. As a result, it was confirmed that the HK activity involved in phosphorylation can be estimated from the developed assay system in real time by tracking the ADP/ATP ratio change, rather than the enzyme substrate, glucose.

### Monitoring dephosphorylation in a kinase reaction

**Fig. 3:**
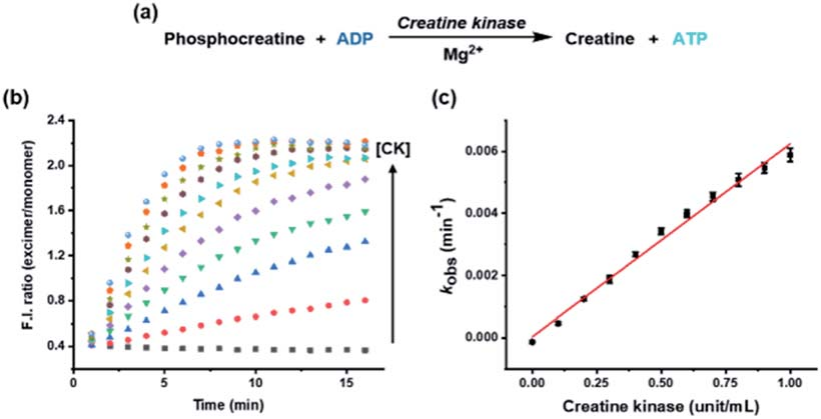
(a) Enzymatic reaction formula of CK associated with dephosphorylation; (b) fluorescence intensity ratios of excimer (F_476_) and monomer (F_376_) emissions over time for the pyrene-DPA-Zn^2+^ complex with various concentrations of CK (0 to 1 unit per mL) in buffer solution (HEPES, 20 mM, pH 7.4); (c) plot of *k*_obs_ against concentration of CK. [pyrene-DPA-Zn^2+^ complex] = 20 μM, [ADP] = 10 μM, [Mg^2+^] = 80 μM, [phosphocreatine] = 0.75 mM, *λ*_ex_ = 341 nm.

Although most kinases are involved in phosphorylation of substrates, some are used for dephosphorylation, with transfer of the phosphate group from the substrate to ADP. To establish a more versatile kinase assay method, it is also crucial to analyze the kinases associated with dephosphorylation. Herein, CK, which transfers a phosphate group from phosphocreatine to ADP, was exploited as an example enzyme to show the method's application for the dephosphorylation process (Fig. 3a).^26^ Progression of the CK reaction caused a decrease in the ADP/ATP ratio, the opposite result to that with HK, which could be inferred from the decreased monomer emission at 376 nm and increased excimer emission at 476 nm (Fig. S4†). The change in ratiometric fluorescence was proportional to the concentration of CK from 0 to 1 unit, and the rate constant, which was estimated from the fluorescence change, displayed a linear correlation with the concentration of CK (Fig. 3b and c). Therefore, the developed kinase assay system can be applied to analyze dephosphorylation as well as phosphorylation in real time without additional assistance, such as a modified substrate or cascade enzyme reaction.

Most typical kinase assays except for recently reported research, which assayed both hexokinase and pyruvate kinase as a sequential monitoring,^31^ have focused on the analysis of only mono-directional changes, either phosphorylation or dephosphorylation, although the methods were based on changes in ATP and ADP and then extended for several kinases.^17,20^ However, considering the reversibility of reactions exhibited by kinases, it is necessary for a general kinase assay to analyze the bidirectional changes between ATP and ADP that indicate both phosphorylation and dephosphorylation. The developed assay method in this study provided evidence for the effective utilization of bidirectional changes in kinase reactions, and consequently, the results are suggested as a general method for versatile small molecule kinase assays.

### Inhibitor assay for the kinases

**Fig. 4:**
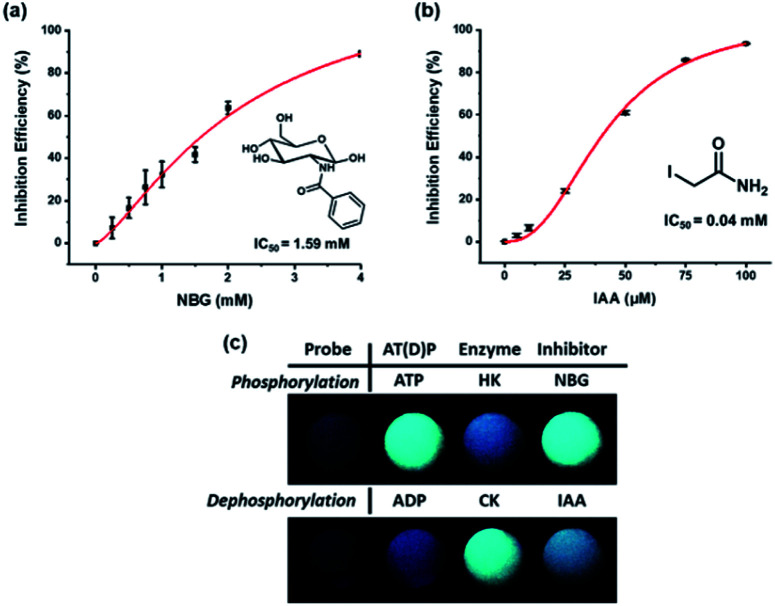
Inhibition efficiency for (a) HK and (b) CK with various concentrations of NBG and IAA, respectively, in buffer solution (HEPES, 20 mM, pH 7.4) with each substrate; (c) photographs of the pyrene-DPA-Zn^2+^ complex in the presence of kinases and inhibitors. [pyrene-DPA-Zn^2+^ complex] = 20 μM, [ATP] or [ADP] = 10 μM, [Mg^2+^] = 80 μM, [glucose] = 1 mM, [phosphocreatine] = 0.75 mM, [HK] = 1 unit, [NBG] = 5 mM, [CK] = 1 unit per mL, [IAA] = 100 μM, *λ*_ex_ = 341 nm.

The fact that most kinases play an essential role in biological processes makes the development of inhibitors crucial. To demonstrate that the suggested kinase assay method can be used for inhibitor assays, the efficiency of inhibitors for both HK and CK was measured by using the change in ratiometric fluorescence. The inhibition efficiencies of well-known inhibitors of HK and CK, *N*-benzoyl-d-glucosamine (NBG) and iodoacetamide (IAA), respectively, were confirmed against an increased concentration of inhibitor (Fig. 4). The half maximal inhibitory concentration (IC_50_) of the inhibitors estimated with the developed assay system was compared with the reported values (Table S2†). The results correlated without any large differences, indicating that the system was applicable as an inhibitor assay.^38,39^

In addition, rapid and simple screening methods for multiple inhibitor candidates are required in the pharmaceutical field. To achieve this purpose, the microwell plates were photographed upon irradiation with a handheld UV lamp to record the fluorescence changes for the enzyme activity and inhibitor efficiency. As shown in Fig. 4c, during phosphorylation, the excimer emission observed in the presence of ATP was changed to monomer emission by HK activity, and the inhibition of enzyme activity by NBG allowed the probe to maintain the excimer emission. By contrast, CK changed the monomer emission to excimer emission, but monomer emission was maintained in the presence of IAA. It is believed that these results, in which enzyme activity and inhibitor efficiency can be analyzed by the naked eye, will permit the application of the developed system to high-throughput screening of kinase inhibitors.

## Conclusions

In summary, a real-time and versatile small molecule kinase assay system using a ratiometric fluorescence probe was proposed. The developed system, based on monitoring of the interchange of ATP and ADP, can be used for various kinases, regardless of their specific substrate. In addition, the reversible fluorescence change of the ratiometric fluorescence probe is applicable for both phosphorylation and dephosphorylation performed by various small molecule kinases, unlike other methods, which have only focused on mono-directional analysis of kinase reactions. As a proof of concept, the activities of HK and CK were analyzed as examples of phosphorylation and dephosphorylation, respectively, and the assay system was also used for inhibitor assays. In addition, the fluorescence change was observable with the naked eye, demonstrating the applicability of this method to high-throughput screening. Overall, although a limitation remains that is difficult to apply in cellular environment containing ATP and ADP, the proposed system provides a general method that can be extended to various kinase and inhibitor assays.

## Conflicts of interest

There are no conflicts to declare.

## Supplementary Material

RA-011-D1RA01547H-s001
