# Supplementary material for: Versatile small molecule kinase assay through real-time, ratiometric fluorescence changes based on a pyrene-DPA-Zn2+ complex
Source: RSC Adv. 2021 Mar 10;11(17):10375–80. doi: 10.1039/d1ra01547h (PMC8695712; doi:10.1039/d1ra01547h)
Supplement: RA-011-D1RA01547H-s001 [file RA-011-D1RA01547H-s001.pdf]

Electronic Supplementary Material (ESI) for RSC Advances.
This journal is © The Royal Society of Chemistry 2021

Electronic Supplementary information

Versatile small molecule kinase assay through real-time, ratiometric fluorescence changes based on a pyrene-DPA-Zn^{2+} complex

Jihoon Kim,‡ Jinyoung Oh‡ and Min Su Han*

Department of Chemistry, Gwangju Institute of Science and Technology (GIST), Gwangju 61005, Republic of Korea

* Corresponding author. E-mail: happyhan@gist.ac.kr

Fluorescence spectra of pyrene-DPA-Zn^{2+} complex with ATP and ADP each

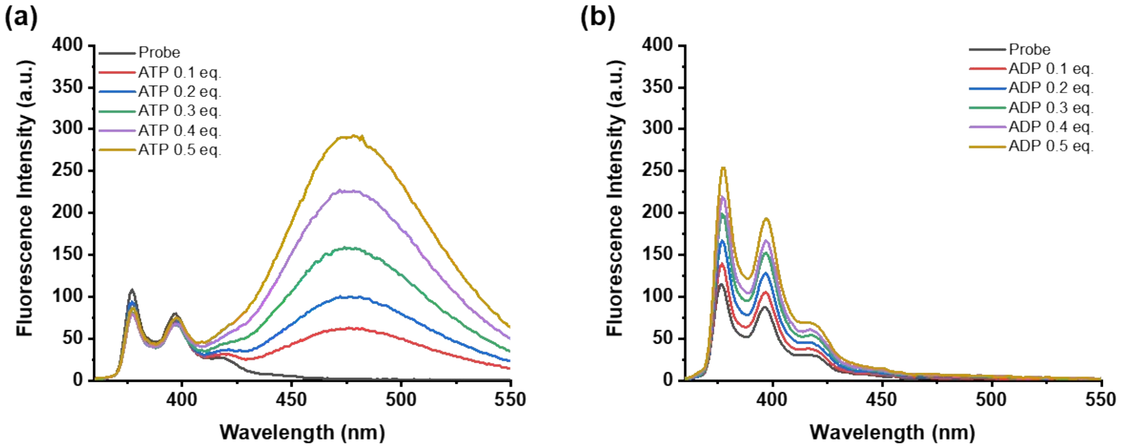

Fig. S1 Fluorescence spectra of pyrene-DPA-Zn^{2+} complex with various equivalents of (a) ATP and (b) ADP in buffer solution (HEPES, 20 mM, pH 7.4). [pyrene-DPA-Zn^{2+} complex] = 20 μM, [ATP] or [ADP] = 0, 2, 4, 6, 8, 10 μM, λex = 341 nm.

Fluorescence spectra change of pyrene-DPA-Zn^{2+} complex by hexokinase

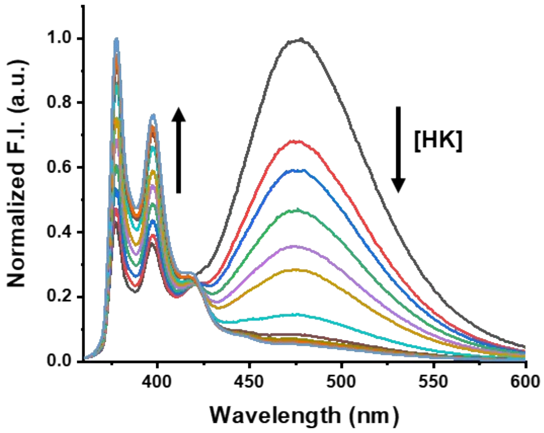

Fig. S2 Fluorescence spectra of pyrene-DPA-Zn^{2+} complex with various units of hexokinase in buffer solution (HEPES, 20 mM, pH 7.4) containing ATP, Mg^{2+}, and glucose. [pyrene-DPA-Zn^{2+} complex] = 20 µM, [ATP] = 10 µM, [Mg^{2+}] = 80 µM, [glucose] = 1 mM, [hexokinase] = 0, 0.1, 0.2, 0.3, 0.4, 0.5, 0.6, 0.7, 0.8, 0.9, 1.0 units, λex = 341 nm.

Enzymatic parameters of hexokinase

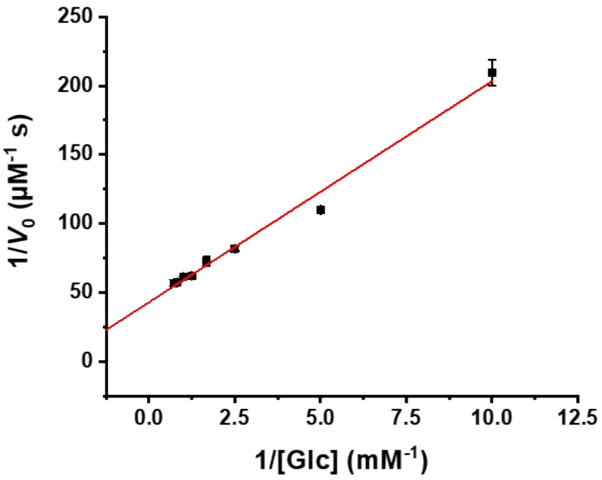

Fig. S3 Lineweaver–Burk plots for activity of hexokinase *versus* concentration of glucose obtained from ratiometric fluorescence changes by pyrene-DPA-Zn^{2+} probe.

Table S1 Enzymatic parameters obtained from our method and conventional method.

Method	Substrate	K_m (mM)	V_{max} (μmol min^{-1} mg^{-1})
This study	Glucose	0.367	0.665
Reported[S1]	Glucose	0.12	0.730

Fluorescence spectra change of pyrene-DPA-Zn^{2+} complex by creatine kinase

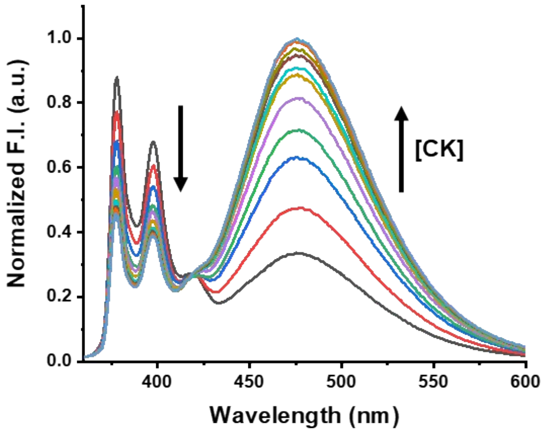

Fig. S4 Fluorescence spectra of pyrene-DPA-Zn^{2+} complex with various units of creatine kinase in buffer solution (HEPES, 20 mM, pH 7.4) containing ADP, Mg^{2+} and phosphocreatine. [pyrene-DPA-Zn^{2+} complex] = 20 µM, [ADP] = 10 µM, [Mg^{2+}] = 80 µM, [phosphocreatine] = 0.75 mM, [creatine kinase] = 0, 0.1, 0.2, 0.3, 0.4, 0.5, 0.6, 0.7, 0.8, 0.9, 1.0 units, λex = 341 nm.

Estimated Inhibition constant for hexokinase and creatine kinase by the developed system

Table S2 Inhibition constant of N-benzoyl-D-glucosamine (NBG) for HK and iodoacetamide (IAA) for CK.

Inhibitor	IC_{50} (mM)	IC_{50} (mM) from ref[S2]
NBG	1.59	7.00
IAA	0.04	0.03

Reference

[S1] (a) N. J. Berthels, R. R. C. Otero, F. F. Bauer, I. S. Pretorius, and J. M. Thevelein*, Appl Microbiol Biotechnol*, 2008, **77**, 1083.

[S2] (a) M. Willson, I. Alric, J. Perie and Y. H. Sanejouand, *J. Enzyme Inhibition*, 1997, **12**, 101; (b) C. J. Xu, W. E. Klunk, J. N. Kanfer, Q. Xiong, G. Miller and J. W. Pettegrew, *J. Biol. Chem.*, 1996, **271**, 13435.

Compound characterization

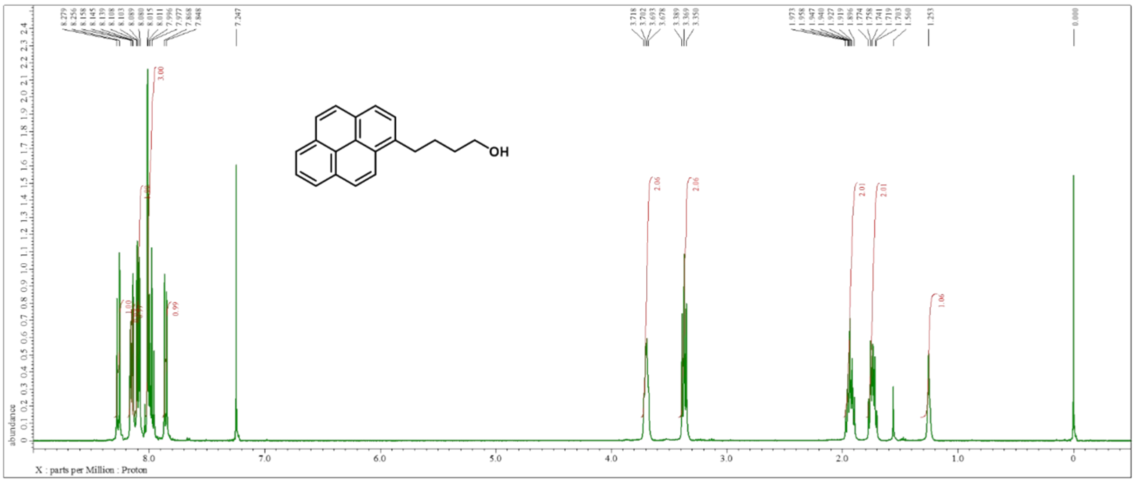

Fig. S4 ^{1}H-NMR of 1-pyrenebutanol, compound **1** in $CDCl_3$.

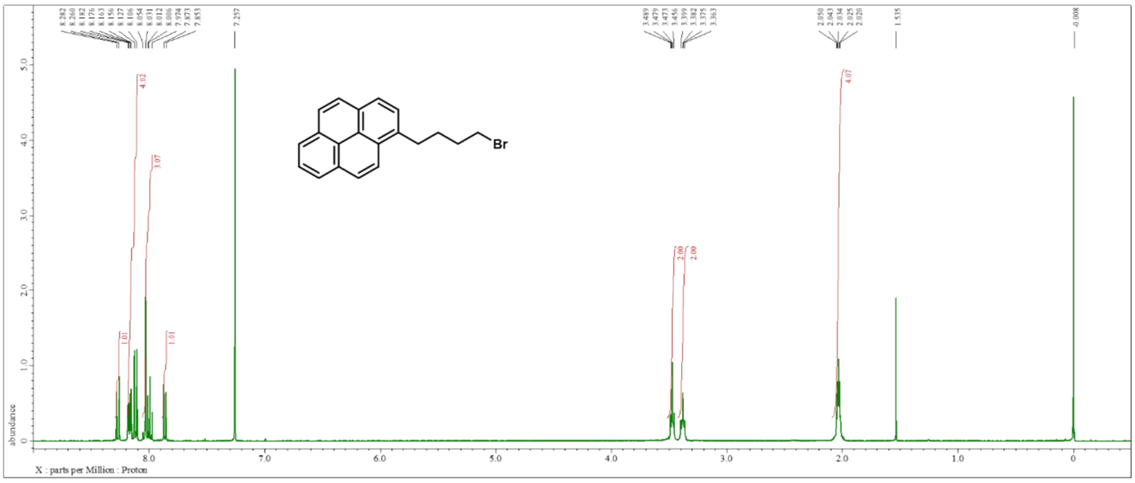

Fig. S5 ^{1}H-NMR of 1-(4-bromobutyl)pyrene, compound **2** in $CDCl_3$.

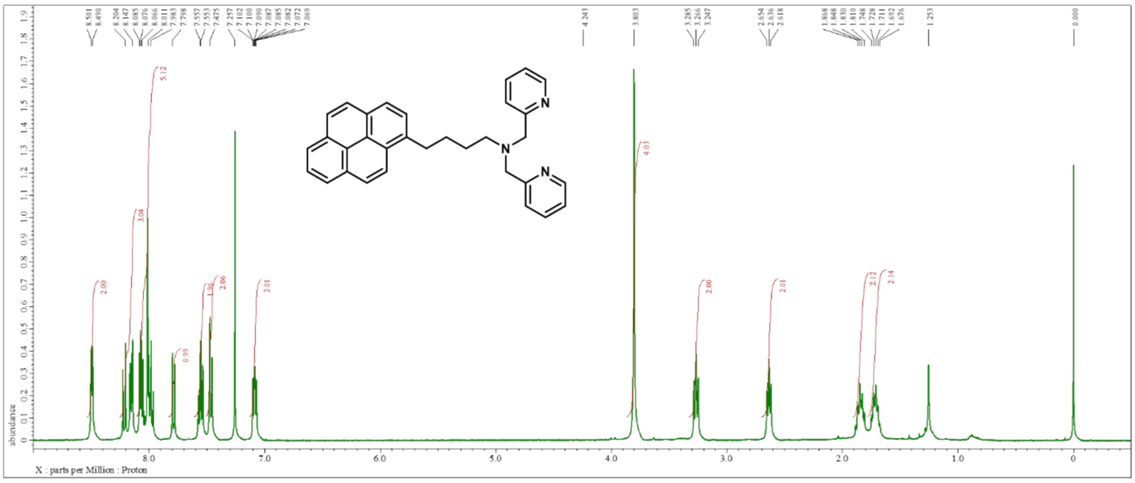

Fig. S6 ^{1}H-NMR of [(2,2'-dipicolyamino)methyl]pyrene, compound **3** in $CDCl_3$.
